# Guillain-Barre Syndrome in a Patient With COVID-19 Infection

**DOI:** 10.7759/cureus.17052

**Published:** 2021-08-10

**Authors:** Fady Sidhom, Harminder Sandhu

**Affiliations:** 1 Internal Medicine, Howard University Hospital, Washington, USA

**Keywords:** guillain-barre syndrome (gbs), covid 19, sars-cov-2 (severe acute respiratory syndrome coronavirus -2), neurological effects of coronavirus, guillain barrè syndrome

## Abstract

Since the identification of SARS-CoV-2 or COVID-19 in Wuhan, several case reports have proposed a possible association between Guillain-Barre syndrome (GBS) and COVID-19. We describe a case of a 59-year-old female who initially presented with shortness of breath, tested positive for COVID-19, and subsequently developed acute hypoxic respiratory failure requiring mechanical ventilation. Her recovery was complicated by acute polyneuropathy. Clinical exam and lumbar puncture showing an albuminocytologic dissociation were consistent with GBS. The prevalence and association of GBS following COVID-19 infection require further research to understand the short and long-term neurological effects of COVID-19, as well as the management of these various neurological manifestations.

## Introduction

Guillain-Barre syndrome (GBS) is an autoimmune disorder where the immune system attacks the peripheral nerves through the mechanism of molecular mimicry. GBS is typically preceded by a viral or bacterial illness. The pathogenesis of GBS is believed to be through an antecedent infection that leads to an immune response that cross-reacts with peripheral nerves through a process called molecular mimicry. Campylobacter jejuni infection is the most common precipitant, but less common etiologies include Cytomegalovirus, Epstein-Barr virus, Mycoplasma pneumoniae, Influenza-like illnesses, HIV, and possibly COVID-19. A case-control study of 102 GBS participants by Rees JH et al. found that 26 percent of individuals had evidence of recent Campylobacter infection, compared with one to two percent of control participants [1]. Other studies have shown the association of Campylobacter and GBS to be as high as 60 to 70 percent of cases depending on the type of GBS [2]. While Campylobacter remains the most common etiology of antecedent infection for GBS, many viral illnesses have been hypothesized to be associated with the development of GBS, including the novel COVID-19. 

Several case reports have been published that have associated COVID-19 and GBS. A case report by Griffin JW et al. found 12 published cases of COVID-19 associated GBS cases [3], and another report published by Rahimi K also found 31 cases of GBS associated with COVID-19 as of August 2020 [4]. Contrary to these case reports, a UK epidemiological and cohort study published in February 2021 by Keddie S et al., found that although it couldn't rule out an association between GBS and COVID-19, that GBS incidence had fallen during the pandemic [5]. The study attributed this drop in prevalence as a result of lockdown measures reducing the transmission of pathogens such as Campylobacter and respiratory viruses that typically cause GBS. Similar to other coronaviruses such as severe acute respiratory syndrome coronavirus 2 (SARS-CoV) and the Middle East respiratory syndrome (MERS-CoV), COVID-19 causes a number of neurological symptoms, and since other coronaviruses have been associated with GBS, it is reasonable to study further the association of COVID-19 and GBS. The case presented highlights a patient with no residual neurological defects that developed GBS with no other known preceding infectious etiologies besides COVID-19. This case also shows the significant clinical cascade that COVID-19 can trigger and its impact on morbidity.

## Case presentation

**Figure 1:**
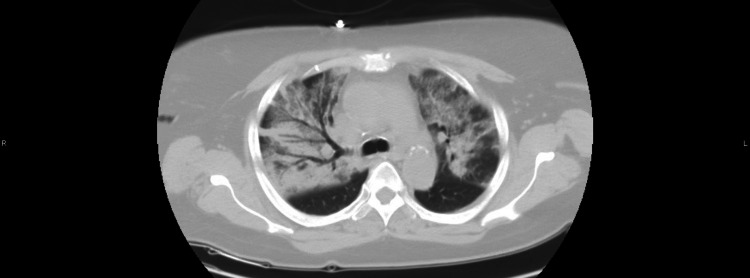
CT Chest w/o contrast image 1

**Figure 2:**
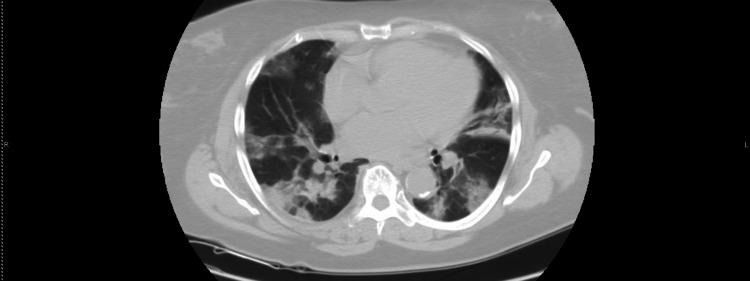
CT Chest w/o contrast image 2

The patient is a 59-year-old female with a past medical history of hypertension, hyperlipidemia, cerebral vascular accident with no residual weakness, and type II diabetes mellitus. She presented with shortness of breath of two-day duration. Her shortness of breath began only with exertion and progressed to dyspnea at rest as well. The patient also had a productive cough of white sputum that began two days prior, as well as a headache and generalized body aches. The patient also endorsed two episodes of watery diarrhea. Of note, the patient reported two possible recent COVID-19 exposures: one being in an out-of-state wedding with a large number of people one month prior, and the second being in a dinner party about a week prior where an attendee was previously diagnosed with COVID-19 pneumonia. Physical exam showed an obese woman in mild respiratory distress requiring a high-flow nasal cannula with diffuse rales bilaterally on auscultation of the chest. The neurological exam was unremarkable. Initial labs were notable for mild azotemia [blood urea nitrogen (BUN)=38, creatinine (Cr)=2.3 (unknown baseline)], serum glucose=335, elevated COVID inflammatory markers [D-dimer=1.1, c-reactive protein (CRP)=21.7, lactate dehydrogenase (LDH)=491, ferritin=55.7], and anemia with a hemoglobin=8.6 and hematocrit=27.9. CT chest without contrast was significant for bilateral central and parenchymal opacities that have the typical appearance of COVID-19 pneumonia as seen in Figure 1 and Figure 2. COVID-19 polymerase chain reaction (PCR) test was positive and the patient was admitted directly to the ICU for management of acute hypoxic respiratory failure secondary to COVID-19 pneumonia.

In the ICU, the patient was initially maintaining her saturation on a high-flow nasal cannula, but within a few hours, she began desaturating to the 40s while on a fraction of inspired oxygen (FiO2) of 1.00. The patient was initially reluctant to intubation but due to worsening respiratory distress, the patient consented and was subsequently intubated. The patient also initially required vasopressor support with Levophed but was weaned off promptly and blood pressure was well controlled with the resumption of home antihypertensives. She was started on thiamine, zinc, and vitamin C and D and completed a 10-day course of intravenous dexamethasone. The patient also completed a seven-day course of Ceftriaxone and a five-day course of Doxycycline to cover for community-acquired pneumonia. The patient received two doses of Remdesivir, but further doses were held due to the development of acute kidney injury (AKI). Nephrology was consulted for management of AKI and recommended only conservative management with no need for hemodialysis. Cardiothoracic surgery was also consulted to evaluate for the use of extracorporeal membrane oxygenation (ECMO) but deemed that the patient did not require ECMO during her ICU course. Serial blood cultures showed no growth but a urine culture grew pan-sensitive Pseudomonas and the patient completed an additional five-day course of Meropenem. The ICU course was complicated by an acute hemorrhage where the patient lost approximately one liter of blood from the left femoral site, where an arterial line was previously placed. The patient was subsequently bolused with 1L of normal saline and transfused two units of packed red blood cells. Despite no more visible additional bleeding from the femoral site, bloodwork showed persistent drops in hematocrit. The patient required intermittent transfusions to keep her hemoglobin at 7.0. Ultrasound imaging ruled out a hematoma and the patient remained on prophylactic anticoagulation. The patient's oxygen requirements gradually came down and on day 14, the patient was extubated to a venturi mask. The following day, the patient was deemed stable for transfer out of the ICU to the medical floors.

On the medical floors, the patient was weaned off the venturi mask and placed on a nasal cannula. Following extubation, the patient was noted to only nod her head in response to questions but remained non-verbal. She was also unable to move her upper or lower extremities with the exception of wiggling her toes when prompted. Neuro exam revealed symmetrical quadriparesis and absent deep tendon reflexes. CT head imaging showed no acute intracranial pathology, and only noted mild cerebellar atrophy. Due to the patient's immobility and high risk of deep vein thrombosis (DVT), heparin was increased from prophylactic dosage up to 7500U Q8H. Ultrasound doppler of the upper extremities found a floating clot in the right internal jugular (RIJ), the site of a previous central line, and the patient was transitioned from heparin to therapeutic Lovenox. Neurology was consulted to evaluate the quadriparesis and recommended electrodiagnostic studies as well as a lumbar puncture (LP) to evaluate for GBS. Electromyography was ordered but was never completed due to limited hospital resources related to the pandemic. Lovenox was held and a lumbar puncture was performed. LP fluid analysis showed WBC=1 (0-5), RBC=3 (0-20), total protein=50 (15-45), and glucose=112 (40-80), consistent with an albuminocytologic dissociation. A test for glycoprotein Q1 (GQ1) antibodies was also ordered and was negative. Intravenous immune globulin (IVIG) was ordered for management of GBS, but treatment was delayed due to unavailability in the pharmacy. The patient's weakness began to improve from a muscle strength of 1/5 to 2/5 in all extremities while awaiting IVIG administration. Shortly after on day 27 of her hospital course, routine labs showed several new abnormalities including leukocytosis, hyperkalemia, elevated BUN and creatinine, and a significant drop in hemoglobin and hematocrit (H/H) from 8 to 5.5gm/d. Repeat labs were ordered to confirm the sharp changes in the patient’s labs. On examination, the patient was found to have an asymmetrically swollen right hip. An ultrasound was ordered to rule out bleeding into the groin but shortly after, a rapid response was called for acute change in mental status, tachypnea to the 40s, and severe hypotension. The patient was bolused with fluids but continued to decline rapidly. The patient was subsequently intubated for respiratory distress and transferred to the ICU. Shortly after arrival to the ICU, the patient was found to be pulseless with a rhythm of asystole. A cardiac arrest code was called and high-quality chest compressions were started. During the code, the patient's next of kin (NOK) was reached by phone and after discussion of the patient’s prognosis, the family requested for cardiopulmonary resuscitation to be stopped, and the patient subsequently expired. An autopsy was offered to the patient’s family after counseling but the family declined.

## Discussion

The case presented shows a patient that despite recovering from the initial respiratory effects of COVID-19, ultimately suffered from severe neurological complications that may have resulted in her death. Current management of COVID-19 rightfully focuses on the respiratory system, as the virus primarily attacks the lungs. But as this case presents, the focus should also be directed to the neurological manifestations of the virus. A review published by Rahimi K evaluated a series of case reports to explore the relationship between GBS and COVID-19 [4]. The review found that the typical profile is of elderly men with a mean age of approximately 57. Of note, the review did find a case report of a child developing GBS following COVID-19 infection. The most common neurological presentation in patients was weakness in the limbs and acute quadriparesis, similar to our patient who experienced weakness in all four limbs following her ICU stay. The mechanism of action for COVID-19 leading to GBS is believed to be through the virus causing inflammation with interleukin-6 (IL-6) production that leads to an inflammatory cascade, which ultimately causes damage to the peripheral nervous system. 

The progression of neurological symptoms in COVID-19 was reviewed by Rahimi K, who found that the mean time from COVID-19 symptoms to GBS symptoms was 11.92 ± 6.20 days [4]. In our case, the time from COVID-19 diagnosis to neurological symptoms was shortly after extubation - around day 14 - which is within the standard deviation specified in the study. Although it should be noted the timing, in this case, can not be completely ascertained due to the possibility of ICU-related weakness from our patient’s prolonged intubation. GBS is a clinical diagnosis that is supported by cerebrospinal fluid (CSF) analysis and electromyography studies. While our patient did not have supportive electromyography studies, her clinical presentation and CSF studies were consistent with GBS, and electromyography is not required to make a diagnosis of GBS. Also while the possibility of critical care weakness exists in this patient, the physical exam findings of absent deep tendon reflexes and CSF findings were more characteristic of GBS. Likewise, the possibility of ICU-related weakness masking the effects of GBS neuropathy exists, and this can make the diagnosis challenging for clinicians until a patient regains their strength following prolonged ICU stays. This can cause delays in identifying and diagnosing GBS in critically ill COVID-19 patients and requires a high degree of clinical suspicion on the part of clinicians. 

A case report published by Dufour C et al. [6] noted that GBS and Miller Fisher syndrome (MFS) are emerging as a known consequence of COVID-19, and describes a case of a patient that tested positive for the anti-GQ1b antibody following COVID-19 infection. In our case, the patient tested negative for the anti-GQ1b antibody, but the report mentions that there is a lack of positive gangliosidosis-1 (GM1) or GQ1b antibodies cases so far and this could point to different pathophysiology of pre-pandemic and pandemic cases of GBS. The difference in pathophysiology of cases could be the result of a novel target for molecular mimicry. And a case report by Kajani S et al. [7], noted that a lack of GQ1B could provide indirect proof that COVID-19 is probably the preceding infection of MFS cases. More data and cases are required to fully understand the relationship between the ganglioside antibodies and COVID, but the negative antibody in our case provides more support for COVID-19 as a possible etiology for GBS. 

Researchers in Spain conducted a large-scale study identifying GBS in emergency departments during the pandemic [8]. They found that the relative frequency and standard incidence were higher in COVID-19 patients compared to non-COVID-19 patients in a two-month peak of the pandemic. Although this was a large study with over 1.4 million patients across 152 emergency departments (ED), the study identifies several limitations including the small number of total GBS cases identified (11 in COVID-19 patients and 33 in non-COVID-19 patients) and the possibility of underdiagnosing GBS in the ED due to severe COVID-19 patients being rapidly intubated and mild cases not even presenting to the ED. Overall, the researchers felt that although more studies and case reports are required, close attention should be paid to the relationship between COVID-19 and GBS.

## Conclusions

Despite the novel nature of COVID-19 and the ongoing research of the virus, a growing number of case reports have identified a possible association with GBS. While no cohort or epidemiology studies have confirmed an association between GBS and COVID-19, more research is required to determine causality. As research continues around the coronavirus, it’s important to identify clinical associations that can ultimately alter management. Due to previous coronaviruses such as SARS-CoV and MERS-CoV being linked to developing GBS, special attention should be paid to the possible causative effect of COVID-19. It’s also possible that as the prevalence of COVID-19 increases that the number of GBS-related cases also increases concurrently. While further research is required to establish a causal relationship between COVID-19 and GBS, close attention should be paid to the novel virus as a possible etiology.
